# Reviving a Skewed Dyskinetic Scapula Secondary to Ventral Exostosis: A Case Report

**DOI:** 10.7759/cureus.102928

**Published:** 2026-02-03

**Authors:** Santhosh K Meiyappan, Gummalla M Reddy, Narendran Pushpasekaran, Hari Sivanandan

**Affiliations:** 1 Orthopaedics and Traumatology, Vinayaka Mission's Kirupananda Variyar Medical College and Hospital, Vinayaka Mission's Research Foundation (Deemed to be University), Salem, IND; 2 Orthopaedics, Vinayaka Mission's Kirupananda Variyar Medical College and Hospital, Vinayaka Mission's Research Foundation (Deemed to be University), Salem, IND

**Keywords:** dyskinetic shoulder, extended periscapular approach, osteochondroma, snapping scapula, trapezius reflection

## Abstract

Exostosis or osteochondroma of bone rarely arises in the scapula, and when located in the ventral aspect of the scapula, it disrupts the scapulothoracic biomechanics. Another concern is that, unlike the involvement of other sites, the morphology of the cartilage cap involved here appears distorted due to constant friction over the ribs, making the assessment of malignancy changes difficult through routine investigations. We report the case of a 23-year-old man who presented with a mispositioned left scapula and dyskinetic scapulothoracic movement that disrupted overhead activities. Magnetic resonance imaging (MRI) showed a 5 × 4 cm osteochondroma in the ventral surface of the scapula, with distorted and irregular cartilage cap, risking features of sarcomatous changes. The mass excised through an extensive periscapular approach showed mature osteochondroma without sarcomatous changes despite cartilage irregularity. The scapulothoracic rhythm was restored without any surgical site morbidity. This case study highlights the spectrum of symptomology produced by ventral surface scapular osteochondroma and the surgical methods to excise the osteochondroma to restore scapulothoracic rhythm. We reiterate that excision biopsy and histopathological examination of the distorted cartilage cap play an important role in the management of ventral surface scapular osteochondroma, along with reviving the normal scapulothoracic movement.

## Introduction

Osteochondroma or bone exostosis is a common benign tumor of the bone located around the metaphysis of long bones, especially the knee and shoulder. However, involvement of flat bones like the scapula is rarely reported in the literature [1]. Exostosis arising from the ventral surface of the scapula is of significant interest due to its unique location in a pseudo joint and disturbances in the scapulothoracic rhythm [2]. Hence, symptoms range from cosmesis, snapping, winging, and dyskinesis of the scapula [2-4]. Another concern is that the cartilage cap is distorted due to the constant movement across the ribs and less reliable features on imaging studies to assess sarcomatous changes [5,6]. This case study describes a varied presentation of a ventral surface scapular osteochondroma with rib impingement, dyskinesis, and late-onset shoulder movement restriction, and exhibited a malformed, irregular cartilage cap. An extended periscapular approach was required to remove the growth from the scapulothoracic joint and restore the scapula position and movement rhythm.

## Case presentation

**Figure 1:**
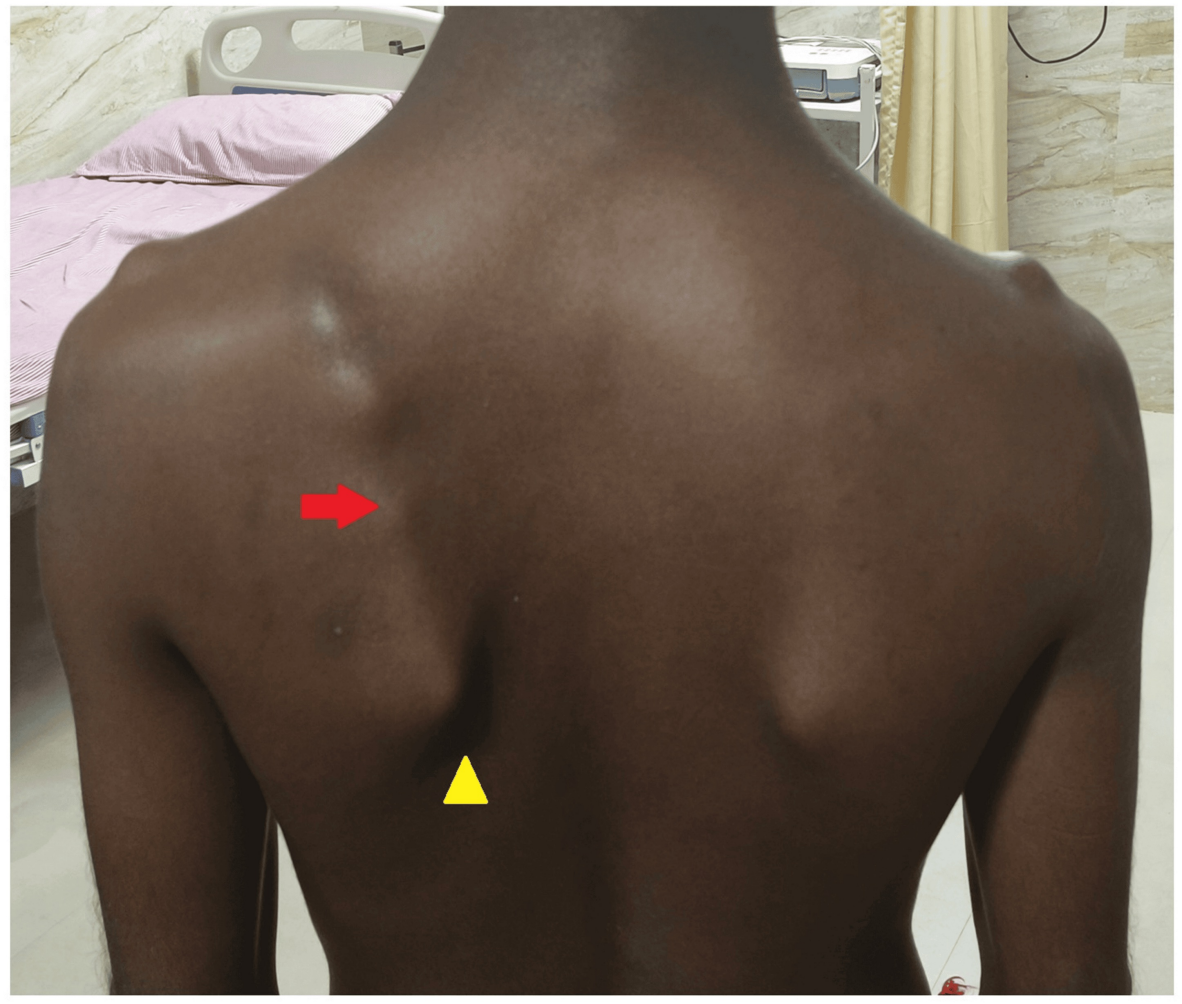
Clinical image showing left scapula malposition and winging The left scapula is protracted, superiorly placed, with a medial border and inferior angle more prominent (yellow arrowhead). The musculature attached to the medial border of the left scapula appears irregular compared to the smooth contours and absent prominences on the right scapula (red arrow).

A 23-year-old man complained of discomfort and crepitus in the left upper back and shoulder during overhead shoulder activities and when lying over the back. Examination revealed an asymmetrical position of the scapula with a prominent medial border, and the inferior angle of the scapula deviated outward and laterally (Figure 1).

**Figure 2:**
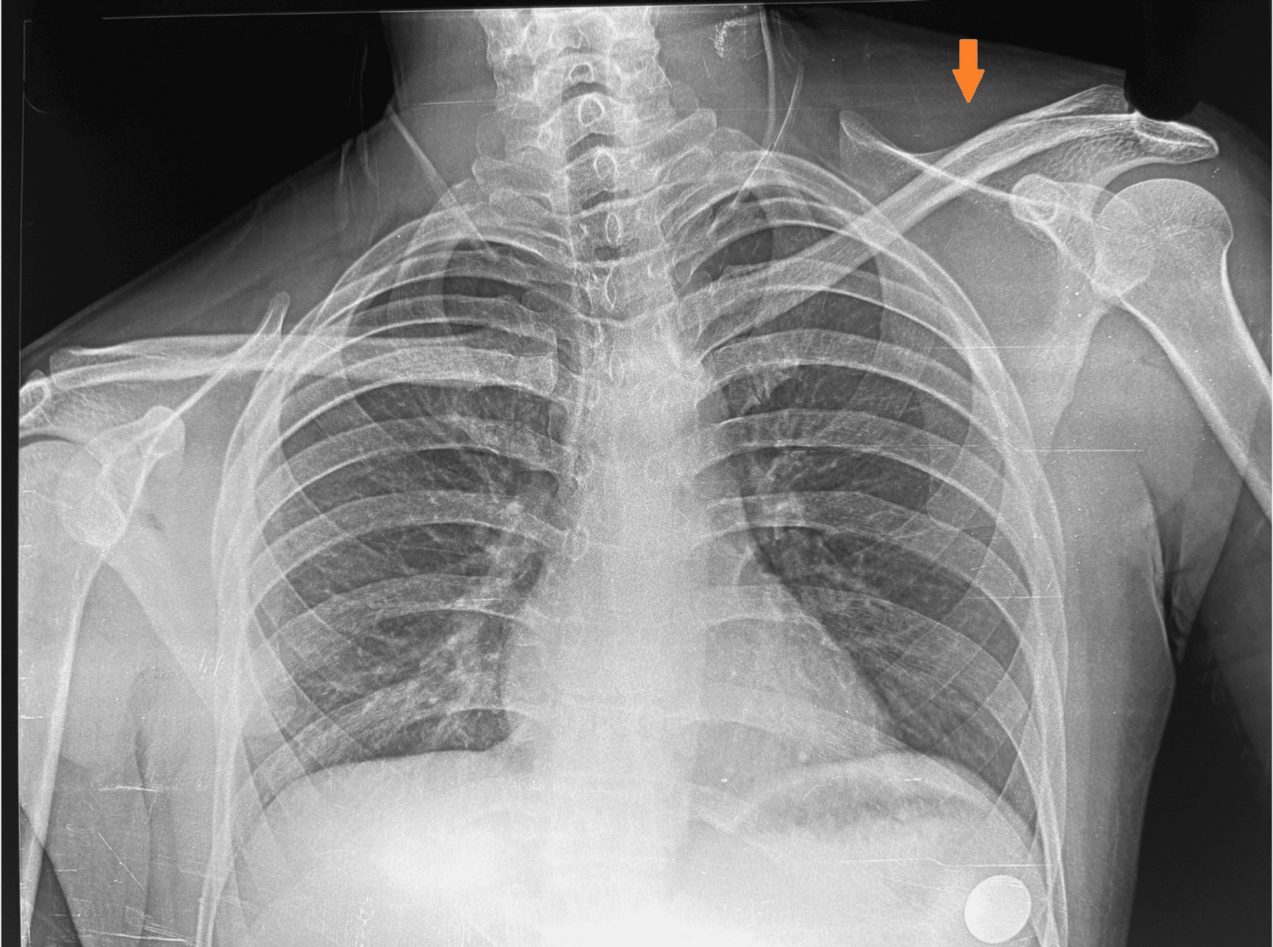
Chest radiograph showing scapula asymmetry The left scapula is malpositioned (orange arrow) and appears elevated and outwardly deviated.

Tenderness and bony prominence were palpated over the middle third of the medial border of the scapula, along with coarse crepitus on protraction and elevation movements. The winging could not be corrected, and shoulder movements, especially abduction and forward flexion, were restricted beyond 90° on attempted scapula stabilization. Chest radiograph showed asymmetrical scapular position (Figure 2).

**Figure 3:**
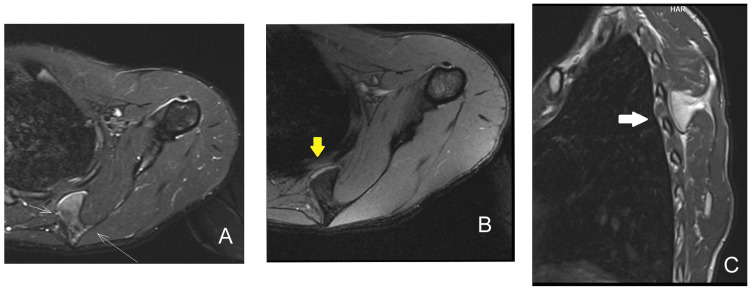
Magnetic resonance imaging of the scapula showing osteochondroma arising from the ventral surface of the scapula impinging on the ribs A: Axillary T2-weighted STIR sequence showing bony growth of size 2.98 × 1.82 cm and the cartilage cap (white thin arrows). B: Axillary section gradient sequence showing cartilage thickness of 1.45 mm at the tip of the bony mass (yellow thick arrow). C: Sagittal T2 sequence showing a pedunculated mass of size 3.32 × 2.48 cm from the scapula impinging on the ribs (white thick arrow). STIR: short-tau inversion recovery

Magnetic resonance imaging (MRI) of the thorax and scapula showed a pedunculated mass with a cartilage cap on the ventral surface of the scapula pressing over the sixth and seventh ribs, favoring an osteochondroma (Figure 3A-3C).

**Figure 4:**
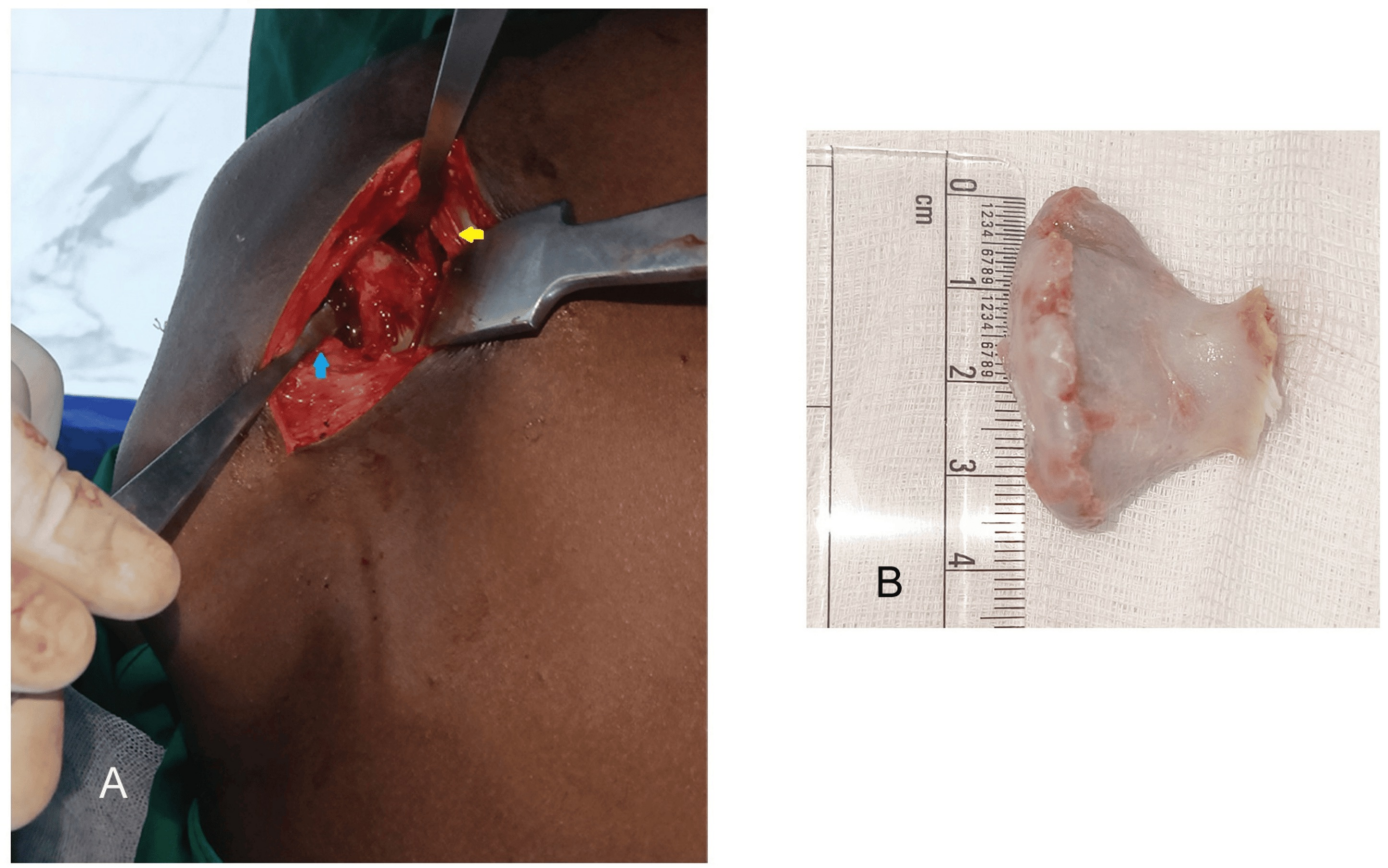
Excision biopsy of the scapular osteochondroma through the medial scapula extended approach A: Intraoperative image showing the patient in prone position, with the arm held in chicken wing position, with vertical skin incision and trapezius reflection superiorly (yellow arrow) and rhomboid major retracted inferiorly (blue arrow). B: Excised bony protrusion of size 3.6 × 4 cm showing irregular cartilage borders.

There were no signs of bursitis or collection in the scapulothoracic space, and the ribs were normal. An excision biopsy was executed through an extended periscapular approach under general anesthesia. The patient was positioned in the prone position, with the shoulder and arm internally rotated such that the medial border of the scapula appeared prominent. A 6 cm vertical incision was made in the medial border of the scapula, starting at the level of the spine of the scapula. The trapezius attachment in the middle third of the scapula was reflected, and with a 10 mm saw blade, the pedunculated mass was removed from its base on the ventral surface of the scapula (Figure 4A, 4B).

**Figure 5:**
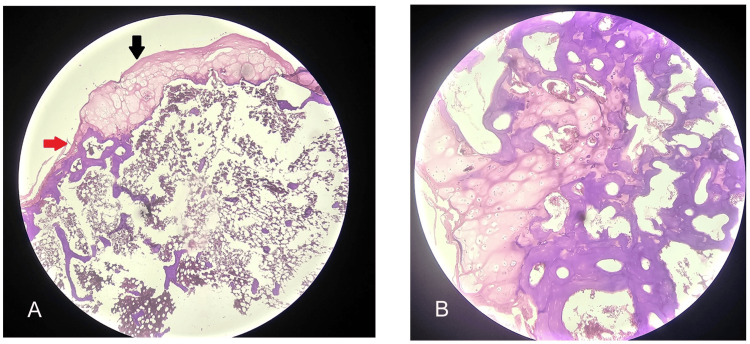
Histopathological examination of the excised specimen from the scapula favored mature osteochondroma and no malignant changes (hematoxylin and eosin stain) A: Scanner view showing the cap that was made of fibrous perichondrium with hyaline cartilage (black arrow), and the underlying area shows transition to enchondral ossification with formation of bony trabeculae having marrow spaces (red arrow). B: 10× view showing hyaline cartilage, enchondral ossification, and marrow spaces.

The excised mass of 4 × 3.6 cm appeared distorted and had an irregular cartilage surface, suspecting malignant features; however, the pathological findings did not show any atypical cellular changes of sarcoma but mature osteochondroma (Figure 5A, 5B).

The scapula symmetry and movement rhythm were restored. There was no recurrence or dyskinesia at the 15-month follow-up period.

## Discussion

Osteochondroma or exostosis of bone is one of the most common benign tumors of bones that occurs at the metaphysis of long bones. Their presence in the flat bones of the pelvis, spine, and scapula is rarely reported [1]. Among the flat bones, exostosis involvement has been least observed in the scapula, with fewer than 30 cases reported in the English literature [2,3,7,8]. The dorsal and the ventral surface of the scapula have exhibited exostosis [9]. Although painless, cosmesis and discomfort on lying down have been the concerning symptoms for dorsal scapular exostosis, the ventral surface involvement presents with pseudo winging of the scapula, snapping sounds, chest wall malformation, and scapula dyskinesis [3,4,7,8]. Limitation of shoulder movements due to mechanical impingement and pain has also been reported in a few instances of ventral osteochondromas, but usually in the terminal range. Kwon et al. reported progressive restriction of abduction and external rotation in a 53-year-old woman for two years secondary to a ventral osteochondroma. Located near the root of the spine of the scapula, the rhomboid muscle function was impaired, and that desynchronized humeroscapular rhythm during overhead movement [2]. Our case featured the same, with difficulty in terminal overhead abduction, and the exostosis of size 3.6 × 4 cm was observed within 3 cm from the root of the spine of the scapula.

Magnetic resonance imaging has a crucial role in assessing the cartilage cap of osteochondromas, which is expected to be smooth and regular. However, in ventral osteochondroma, by virtue of its constant compression and movement over the ribs, the growth surface tends to be flat and irregular, and a thickness of beyond 2 cm has been least reported to suspect malignant changes [6]. Munde et al. observed a similar appearance of ventral scapula osteochondroma in a seven-year-old boy, describing the appearance of the excised mass as cauliflower-shaped and a flattened surface facing the chest wall. MRI was inconclusive in ascertaining the malignant potential. However, histopathology examination was confirmatory of benign osteochondroma [10]. In our case, the excised pedunculated mass appeared with an irregular surface and a distorted peduncle. There were no malignant features on histopathology. To the best of our knowledge, there have been no reports of chondrosarcoma from ventral scapular osteochondroma despite the excised mass being consistently reported as a distorted cartilage cap. However, in view of the abnormal morphology of the cartilage cap in the scapulothoracic joint, we recommend histopathological examination of the lesion to note the benign or malignant features.

The ventral surface of the scapula and the scapulothoracic joint can be approached directly through a vertical incision just over the medial border of the scapula with the patient in the prone position [11,12]. Based on the location and size of the osteochondroma, the reflection of the trapezius and rhomboid major varied and must be attached meticulously to prevent iatrogenic winging of the scapula. Although muscle-sparing approaches such as arthroscopic excision and approach through the triangle of auscultation space have been described, they are less extensive and risk long thoracic nerve damage [13,14]. Prakash et al. excised three cases of ventral scapula exostosis through an intermuscular plane formed by the latissimus dorsi and trapezius that required only minimal reflection of the rhomboid major muscle [13]. However, the utility of this approach was limited to the osteochondromas that were closer to the inferior angle of the scapula. In our case, the pedunculated osteochondroma was located in the middle third and medial border of the scapula near the spine crest, which required reflection of the trapezius and splitting the rhomboid major muscle. The reflected part of the trapezius was reattached by transosseous suturing to the scapula. We did not observe any scapular asymmetrical movements or winging during follow-up. However, caution should be exercised to reflect the trapezius close to the scapular attachment, where the fibrous and tendinous portion is maximum and less muscular.

## Conclusions

Exostosis in the ventral aspect of the scapula presents with a spectrum of symptoms, such as snapping, winging of the scapula, shoulder dyskinesis, rib deformities, frequent discomfort and pain, limitation of terminal abduction and external rotation rarely, and shoulder impingement due to the mechanical effects of mass in the scapulothoracic joint, disrupting the rhythm of the scapula and humerus movements. The constant compression and movement over the ribs distorts the cartilage cap, resulting in irregular surface and shape, and mimicking malignant changes. However, histopathological examination remains benign, and no chondrosarcoma has been reported from ventral exostosis in the literature. Vertical incision over the medial border of the scapula or the medial parascapular approach with trapezius reflection offers an extensive approach for ventral scapula exostosis removal.
